# Spectral control of elastic dynamics in metallic nano-cavities

**DOI:** 10.1038/s41598-017-11099-y

**Published:** 2017-09-06

**Authors:** Henning Ulrichs, Dennis Meyer, Florian Döring, Christian Eberl, Hans-Ulrich Krebs

**Affiliations:** 1 I. Physical Institute, Georg-August University of Göttingen, Friedrich-Hund-Platz 1, 37077 Göttingen, Germany; 2 Institute of Materials Physics, Georg-August University of Göttingen, Friedrich-Hund-Platz 1, 37077 Göttingen, Germany; 3 Laboratory of Micro and Nanotechnology, Paul Scherrer Institut, CH-5232 Villigen PSI, Switzerland; 4 Physikalisch Technische Bundesanstalt, Bundesallee 100, 38116 Braunschweig, Germany

## Abstract

We show how the elastic response of metallic nano-cavities can be tailored by tuning the interplay with an underlying phononic superlattice. In particular, we exploit ultrafast optical excitation in order to address a resonance mode in a tungsten thin film, grown on top of a periodic MgO/ZrO_2_ multilayer. Setting up a simple theoretical model, we can explain our findings by the coupling of the resonance in the tungsten to an evanescent surface mode of the superlattice. To demonstrate a second potential benefit of our findings besides characterization of elastic properties of multilayer samples, we show by micromagnetic simulation how a similar structure can be utilized for magneto-elastic excitation of exchange-dominated spin waves.

## Introduction

From the beginnings of modern solid state physics up to now it has always been essential to understand the excitation spectra of the different subsystems of a solid. Considering semiconductor heterostructures, it is clear that contemporary technology is built-up on the ability to engineer in particular the wave systems for electrons, for instance by tailoring band gaps and defect modes in these gaps. Today, emerging technological concepts utilize other wave phenomena like spin waves in magnonics^1^, or electro-magnetic waves in photonics^2^.

Regarding phonons, artificial materials with periodically varying elastic properties, so called phononic crystals attract scientific curiosity^3^. The conceptually simplest kind of a phononic crystal is a one-dimensional periodic multilayer, also called a phononic superlattice (SL). Superlattices are studied by the optical method of picosecond ultrasonics since more than 30 years^4–9^. Being predominantly surface sensitive, picosecond ultrasonics is especially well-adapted to detect elastic waves localized in the vicinity of the surface. Thus, surface modes in SLs are well known since decades, both theoretically, and experimentally^10–13^. The effect of an acoustically hard capping layer on a SL was studied theoretically Bria *et al*.^14^. Although some reports, as e.g. refs 15 and 16, can be related to this theory, to our knowledge no systematic experimental investigation has been reported. In this article we show experimentally that by varying the thickness of the capping layer, band gaps of a SL can be mapped out. Thus the experiments discussed in this article demonstrate a nano-scale interferometry method for characterization of elastic properties of SLs.

While we demonstrate applicability of this method up to about 400 GHz, we expect that the method can in principle be applied for phonon spectroscopy up to several THz. Note that THz phonons are especially important for heat transport phenomena. Novel effects occur when being able to manipulate a materials structure on the corresponding near atomic-level spatial scale^17^. For instance, it was shown theoretically by Simkin and Mahan^18^, and experimentally by Ravichandran *et al*.^19^ that by reducing the feature size of an epitaxially grown SL down to the atomic scale, coherent self-interference of phonons across several interfaces may occur. Then thermal transport is said to become coherent, in contrast to classical, diffusive transport. Understanding and controlling the physics of phonon heat transport is crucial for the development of novel thermal devices like thermal diodes, thermal transistors and thermal memory^20–23^.

Coherent phonon dynamics is also exploited in hybrid device prototypes like the acoustic laser^24^, or for magneto-elastic excitation of spin dynamics^25–27^. The latter phenomenon bridges into the field of magnonics. There, magnon-phonon interaction are usually rather an unavoidable nuisance related to unwanted dissipation processes, limiting the life time of the spin dynamics. In the outlook chapter of this article we will provide an example for a beneficial, coherent high-frequency magnon-phonon interaction, which can be realized with a structure similar to the one used in our experiment.

## Experimental

The structures under investigation consist of insulating magnesium oxide (MgO), and Yttria-stabilized zirconium oxide (ZrO_2_), which form a periodic multilayer on a silicon substrate, terminated by a MgO layer, finally capped by a metallic tungsten (W) layer. An important parameter for elastic wave propagation is the acoustic impedance *Z* = *ρv*_*L*_, where *v*_*L*_ is the longitudinal sound velocity, and *ρ* the density. While the first two materials have a relatively low acoustic impedance ($31\,\tfrac{{\rm{MPa}}\cdot {\rm{s}}}{{\rm{m}}}$ for MgO, $38\,\tfrac{{\rm{MPa}}\cdot {\rm{s}}}{{\rm{m}}}$ for ZrO_2_), the top most tungsten layer is with $101\,\tfrac{{\rm{MPa}}\cdot {\rm{s}}}{{\rm{m}}}$ acoustically very hard (see Table 1 in supplement).

**Figure 1:**
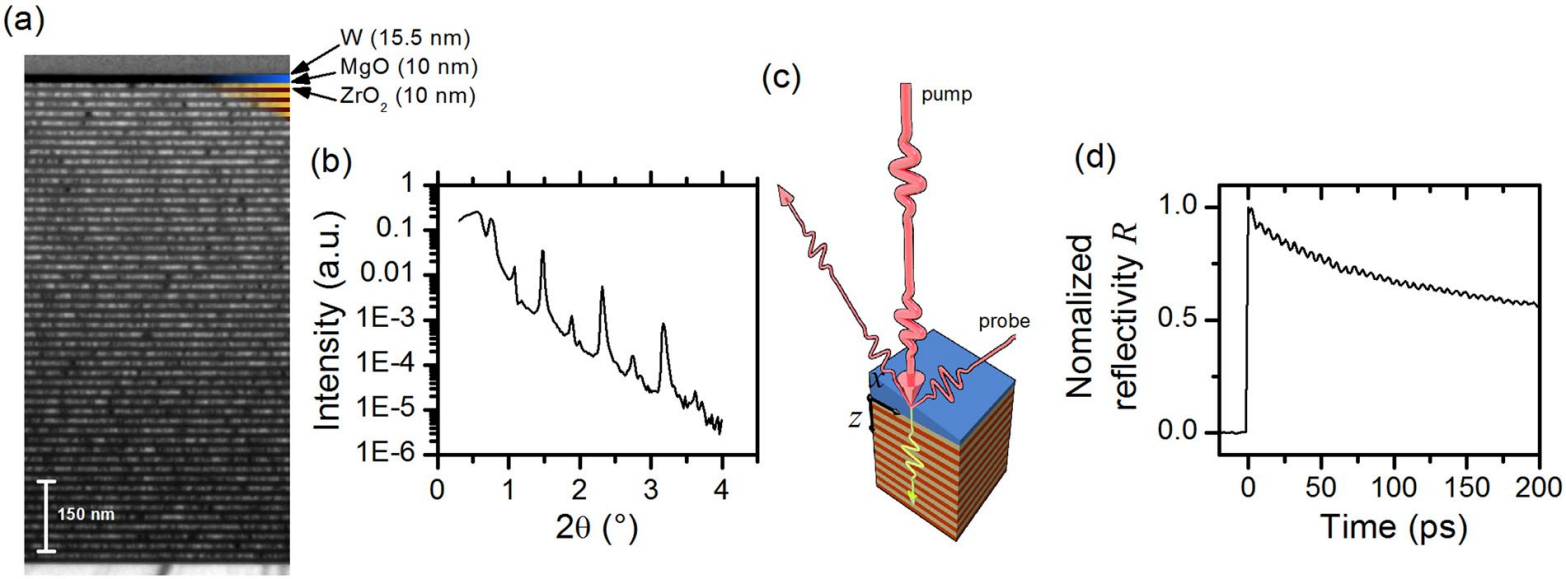
Sample and method. (**a**) TEM image from SL1, obtained in the center of the sample. (**b**) XRR spectrum. (**c**) Sketch of the optical pump-probe experiment. (**d**) Time-resolved reflectivity data, obtained at the same lateral position as the TEM images.

The samples were grown by Pulsed-Laser-Deposition. Automatized control of the deposition system enabled us to deposit a large number of 50 MgO-ZrO_2_ bilayers, as already described elsewhere^28^. In sample SL1 (SL2), each MgO and ZrO_2_ layer has a thickness of 10 nm (5 nm). A lateral thickness gradient of the terminating tungsten layer was achieved by retracting a mechanical shutter during deposition. In SL1 (SL2) the thickness varies between 9 nm (4.5 nm) and 18 nm (9 nm). The samples were investigated by x-ray reflectivity (XRR) and in case of SL1 also by TEM imaging, shown in Fig. 1a,b. From XRR we find a roughness of 0.45 nm for the MgO, and 0.3 nm for the ZrO_2_ layers respectively. The TEM image demonstrates that the ZrO_2_ is amorphous, while the MgO seems to be polycrystalline. In fact, large-angle x-ray diffraction shows that crystallization occurs during TEM lamella preparation by focused-ion-beam technique.

The optical pump-probe experiment is sketched in Fig. 1c. In this stroboscopic method, an intense fs-pulse is split into a strong pump, and a time-delayed, less intense probe part. A slow modulation (mechanical chopper, 800 Hz) of the pump light enables a lockin-based detection of pump-induced changes in the reflected probe light. Being the basic method in the field of picosecond ultrasonics, this approach is well-known for its ability to induce and measure coherent elastic dynamics in the upper microwave frequency range^29, 30^. As a light source we used an amplified Ti:Sapphire fs-pulse laser system. The pump spot was focused to a Gaussian spot with a full-width-half-maximum (FWHM) of 60 *μ*m the probe spot to a FWHM of 20 *μ*m. Due to the smallness of the thickness gradient of about 1 nm per mm, the thickness of the tungsten layer can be considered as locally constant within the laser spot. Probing of the sample at different locations was realized by a motorized micrometer drive, which allows to shift the samples laterally along the *x*-axes (see Fig. 1c). All measurements discussed in the following were performed at an incident optical pump power of *P*_*pump*_ = 130 mW. Figure 1d shows typical time-resolved reflectivity data. One sees a steep increase at time *t* = 0, when pump and probe pulse coincide. This peak is caused by the temperature increase due to absorption. Subsequently the thermal energy diffuses from the surface into the sample and substrate on nano- to microsecond time scales. This process gives rise to a slowly decaying background, which needs to be subtracted from the data, in order to access the superposed oscillations. Then, we apply a Fourier analysis of the remaining signal to obtain quantitative information. Further details can be found in the supplement. Note that in the following discussions we will focus on sample SL1, if not explicitly stated otherwise.

## Results

**Figure 2:**
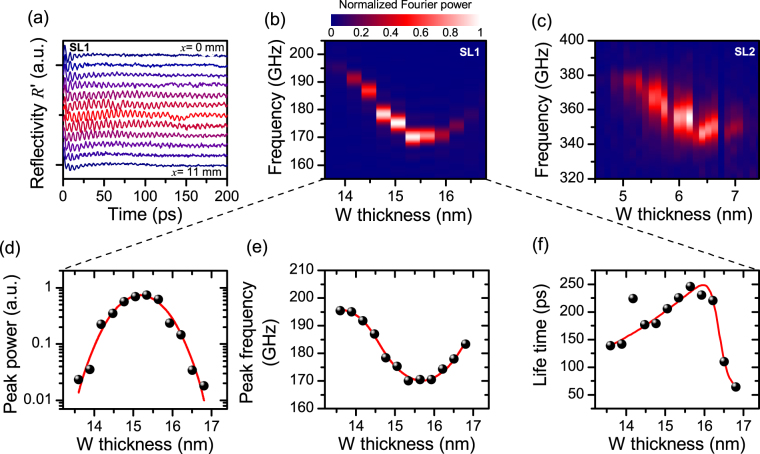
Elastic dynamics on SLs terminated by tungsten wedges. (**a**) Time-dependent reflectivity (background subtracted), obtained at different locations on SL1, and thereby at different W thicknesses, and (**b**) corresponding Fourier power spectra. (**c**) Power spectra, determined from reflectivity measurement from SL 2. (**d**–**f**) Show further analysis of the dynamics found on SL 1. (**d**) Dependence of peak power, (**e**) central peak frequency, and (**f**) life time on the tungsten layer thickness.

Shifting the laser over the sample SL1, we have obtained the reflectivity spectra shown in Fig. 2a. There the tungsten layer has a local thickness between 14 nm and 16.5 nm. These measurements clearly demonstrate that only in the central part of the sample, persistent oscillations could be induced. At thickness lower or larger than the here depicted interval, no signals from coherent elastic dynamics were detected. The relevant parts of the Fourier power spectra corresponding the data shown to Fig. 2a are depicted in Fig. 2b. The experimental data obtained from SL1 was further analyzed by fitting Lorentzian functions to the peaks in the power spectra. The thickness dependences of the thereby derived quantities (central peak frequency *f*_0_, peak Fourier power, and life time $\tau =\tfrac{1}{2{\rm{\Delta }}f}$) are shown in Fig. 2d–f. The peak power displayed in Fig. 2d reveals a Gaussian-like resonance characteristic, with a maximum at 15.5 nm. Simultaneously, the peak frequency decreases between 14 nm and 16 nm. At larger thickness, the frequency increases again (see Fig. 2e). The life time shown in Fig. 2f is with about 250 ps largest at 15.5 nm, and drops for increasing thickness quickly to values of about 50 ps.

Repeating the experiment with sample SL2, the power spectra shown in Fig. 2c were determined. The direct comparison of data obtained from SL1 and SL2 shows that reducing the feature size of the superlattice by a factor of two, obviously doubles the frequency at which the resonance appears, and concomitantly reduces the corresponding thickness of the W layer by a factor of two.

## Analytical model

**Figure 3:**
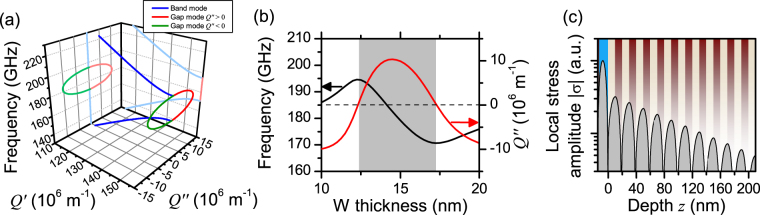
Analytical modelling of elastic dynamics in SL1. (**a**) Band structure diagram showing londitudinal waves in an idealized infinite SL, calculated from solving Eq. (2), and the dispersion of the surface resonance in a terminated SL (closed circle). (**b**) Thickness dependence of frequency and imaginary wave number of the surface resonance. (**c**) Analytically determined stress |*σ*(*z*)| profile of the resonance in the tungsten layer and it’s tail in the SL for *t*_W_ = 15 nm.

In order to understand the experimental results, we will now introduce an analytical model, which is essentially a modification and simplification of theoretical work by Djafari-Rouhani *et al*.^10^. Note that phonons in the microwave frequency range are well-described by elastic continuum dynamics. Furthermore, the laser-induced thermal expansion in negative z-direction primarily excites longitudinal elastic waves. Thus, the equation of motion for the displacement *u*_*z*_ normal to the sample plane, which is relevant for our experiment, reads:

1 $${\rho }_{i}\frac{\partial {u}_{z}}{\partial t}={v}_{i}^{2}\frac{{\partial }^{2}{u}_{z}}{\partial {z}^{2}},$$

where *ρ*_*i*_ is the density, and *v*_*i*_ is the longitudinal speed of sound of layer *i*. The usual boundary condition is that at each interface displacement and stress should be continuous. Before we describe the full system, we will briefly recall that in case of an infinite SL, the solutions of (1) are Bloch-modes, whose dispersion *f*(*Q*) follows from solving Rytov’s equation^13, 31^:

2 $$\cos \,(QL)=\,\cos \,(\frac{2\pi f{t}_{1}}{{v}_{1}})\,\cos \,(\frac{2\pi f{t}_{2}}{{v}_{2}})-\xi \,\sin \,(\frac{2\pi f{t}_{1}}{{v}_{1}})\,\sin \,(\frac{2\pi f{t}_{2}}{{v}_{2}}),$$

where $\xi =\frac{1}{2}\,(\frac{{\rho }_{1}{v}_{1}}{{\rho }_{2}{v}_{2}}+\frac{{\rho }_{2}{v}_{2}}{{\rho }_{1}{v}_{1}})$, and the index 1 stands for MgO and 2 for ZrO_2_. Note that here the wave number *Q* = *Q*′ is strictly real. A part of the dispersion is shown in Fig. 3a. This part covers the first zone-edge band gap, opening at $Q^{\prime} =\frac{\pi }{L}$, with *L* = *t*_1_ + *t*_2_.

In case of a semi-infinite SL with a tungsten layer of thickness *t*_W_ on top, we apply the following ansatz

3 $${u}_{{\rm{W}}}(z)=A{e}^{i{\alpha }_{0}z}+B{e}^{-i{\alpha }_{0}z}$$

4 $${u}_{1}(z)={e}^{iQnL}\{C{e}^{i{\alpha }_{1}z}+D{e}^{-i{\alpha }_{1}z}\}$$

5 $${u}_{2}(z)={e}^{iQnL}\{E{e}^{i{\alpha }_{2}(z-{t}_{1})}+F{e}^{-i{\alpha }_{2}(z-{t}_{1})}\},$$

where $Q=Q^{\prime} +iQ^{\prime\prime} =\frac{\pi }{L}+iQ^{\prime\prime}$, and ${\alpha }_{i}=\frac{2\pi f}{{v}_{i}}$. Eq. 3 is valid for *z* ∈ [−*t*_W_, 0], Eq. 4 for *z* ∈ [*nL*, *nL* + *t*_1_], and Eq. 5 for *z* ∈ [*nL* + *t*_1_, (*n* + 1)*L*] ($n\in {{\mathbb{N}}}_{0}$). These functions describe a resonance in the tungsten layer, which is connected to an exponentially decaying tail inside the multilayer. Demanding that at the surface at *z* = −*t*_W_ the stress vanishes, and continuity of displacement and stress at each interface, we arrive at a system of equations, whose solution yields the frequency *f*_*EWR*_ of the elastic wave resonance and the corresponding imaginary part of the wave number *Q*″. Both quantities depend upon the thickness of the tungsten layer, as Fig. 3b shows. Plotting the resulting dispersion relation *f*_*EWR*_(*Q*″) into the same diagram as the dispersion for the infinite SL (see Fig. 3a), one sees that *f*_*EWR*_(*Q*″) falls into the first zone-edge band gap.

Thus, according to our model, the experimental results can be understood as a coupling between dynamics in the metallic nano-cavity and a surface mode of the SL. Good quantitative agreement between experiments on SL1 and SL2 with the model was achieved by adjusting the speeds of sound for MgO (ZrO_2_) to *v*_*L*_ = 8700 m/s (*v*_*L*_ = 6300 m/s). Describing SL1 with these parameters, the theoretical frequency is bounded between 170 and 194 GHz, and *Q*″ between ±10.3 × 10^−6^ m^−1^. Recall that experimentally, for SL1 the resonance was found between 170 GHz and 195 GHz. Figure 3c shows a profile of the resonance in the cavity for *t*_W_ = 15 nm, and part of its tail in the SL (calculated by evaluating Eqs 3–5). In particular one can see that the reflection at the first interface is strong. This leads to a maximum stress in the center of the tungsten film, and almost vanishing stress at the interface towards the SL. Inside the SL, the evanescent tail displays maxima at each second MgO/ZrO_2_ interface. The exponential decay is clearly seen in the logarithmic plot. We point out, that usually confinement of elastic dynamics to the surface is strongly suppressed, when the top layer has a larger acoustic impedance than the next layer, as it is the case in our samples. Only within band gaps the SL is effectively acoustically harder than the tungsten layer. Note that the response frequency approximately equals that of a free-standing tungsten film, which is $\frac{{v}_{L}}{2{t}_{{\rm{W}}}}$.

These modelling results and interpretations are in perfect agreement with a finite-difference time-domain (FDTD) simulation^32, 33^ of the experiment (see supplement). Note that since neither in this numerical model, nor in the above described analytic model direct losses are taken into account. Both approaches cannot reproduce the complex thickness dependence of the life time (see Fig. 2f), which in turn hints towards an interplay of direct local losses and radiative attenuation in the experiment. The latter increases (*Q*″ → 0) when moving closer to the band edges, whereas direct losses gain importance, the more energy is stored inside the metallic surface layer, which features much larger losses than the insulating constituents of the underlying superlattice.

## Outlook: Spin-wave excitation

In this section we discuss a possible application of our findings: magneto-elastic excitation of exchange-dominated short wave length spin wave modes. Note that it has already been shown by Jäger *et al*.^26^ that surface waves in superlattices terminated by a ferromagnetic layer can excite the ferromagnetic resonance mode. Here we want to show that the concept can be applied to much higher frequencies.

In a ferromagnetic thin film with thickness *t*, the frequency of standing spin wave resonances can be approximated by ref. 34

6 $${f}_{SWR}\approx \gamma \frac{A\pi }{{M}_{s}}\frac{{n}^{2}}{{t}^{2}},$$

where *γ* is the gyromagnetic constant, *M*_*s*_ is the saturation magnetization, *A* is the exchange constant, and *n* is the order of the spin-wave mode. In contrast, the frequency of the elastic resonance which was discussed in the preceding part of this paper approximately scales with

7 $${f}_{EWR}\approx \frac{{v}_{L}}{2t}.$$

Obviously, both dispersions can intersect for a particular thickness of the film. Consider now a film, made out of a material displaying significant magneto-elastic coupling. In an isotropic ferromagnet, the corresponding contribution to the free energy reads^35^

8 $${U}_{mel}=\frac{{B}_{1}}{{M}_{0}^{2}}\,\sum _{ij}\,{M}_{i}{M}_{j}{\varepsilon }_{ij},$$

where *i* and *j* refer to the three Cartesian coordinate components, and ${\varepsilon }_{ij}=\frac{\partial {u}_{i}}{\partial {x}_{j}}$ is the elastic strain. This energy is associated with an effective magnetic field

9 $${H}_{mel,i}=-\frac{\partial {U}_{mel}}{\partial {M}_{i}}.$$

Note that when the strain is caused by an time-varying resonance mode, this field oscillates with the frequency of this wave, representing a high frequency driving field. The coupling efficiency depends on the overlap of the two mode profiles, and on the relative orientation between the dynamic field and the magnetization. Eq. 9 implies that in case of longitudinal elastic dynamics reaching optimal coupling efficiency demands that the film needs to be magnetized at some intermediate out-of-plane angle. Therefore we haven chosen for the following numerical examples 45° for the orientation of the external field *H*_*ex*_. Note that the external field enters the complete expression of the dispersion relation of spin waves^34^. For the sake of simplicity, we have dropped the corresponding terms in Eq. 6. For small enough thicknesses, the *t* ^−2^ term dominates. Nevertheless the external field can be used as an external control parameter, allowing to tune the resonance condition.

**Figure 4:**
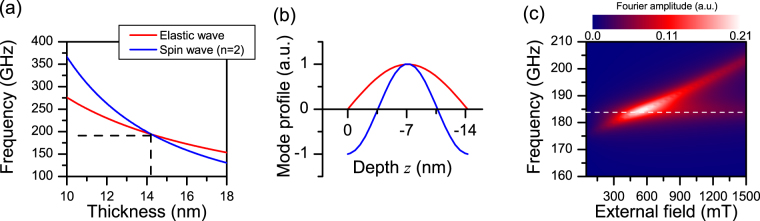
Micromagnetic simulation of elastically driven spin-wave resonance in a magneto-elastic capping layer (CoFeB). (**a**) Thickness dependence of resonance frequencies, calculated by Eqs (6) and (7). Dashed lines indicate the intersection at a thickness of 14.25 nm and a frequency of 184 GHz. (**b**) Normalized profile of elastic surface wave resonance in the CoFeB layer (red curve), used as input for micromagnetic simulation, and resulting normalized profile of the spin wave resonance (blue curve). (**c**) Fourier amplitude spectra of the elastically driven spin dynamics in the center of the CoFeB film as a function of the external field. The dashed line indicates the frequency of the driving elastic wave.

As a concrete example we will now consider a superlattice capped by CoFeB as a ferromagnetic surface layer. The assumed elastic (see Table 1 in the supplement) and magnetic (*μ*_0_ *M*_*s*_ = 1.8 T, *A* = 22 pJ/m) parameters for CoFeB predict an ideal thickness of *t* = 14.25 nm. As shown in Fig. 4a, this thickness is related to a common frequency of 184 GHz of the spin wave resonance and the elastic wave resonance. By coincidence, the first band gap of the SL in sample SL1 covers this frequency. By means of our FDTD method, we simulated ultrafast light-pulse induced dynamics in such a sample, and determined a typical strain amplitude of *ε* = 0.002 in the center of the CoFeB film. With a magnetostriction constant of *λ* = 31 ppm^36^, the resulting magneto-elastic field has a magnitude of *μ*_0_ *H*_*mel*_ = 23 mT. Reflecting our experimental and analytical findings, this field oscillates with frequency 184 GHz, decays exponentially with a time constant of 250 ps, and has a mode profile similar to the one shown in Fig. 3c. We used micromagnetic simulation with the MuMax3 code^37^, and followed the method described in ref. 38 to take into account magneto-elasticity. A three dimensional volume element (32 × 32 × 14.25 nm^3^) was discretized by a proper grid, and extended laterally by periodic boundary conditions.

Fast Fourier transformation of the time-dependent magnetization allows to determine the spin-wave mode profile in terms of FFT amplitude. The spatial dependence of this quantity evaluated at *f* = 184 GHz is shown in Fig. 4b for *μ*_0_ *H*_*ex*_ = 600 mT. This mode profile displays the sinusoidal shape expected from a second order standing spin-wave resonance^34^. To demonstrate the field-controlled tunability of the underlying energy transfer, we show in Fig. 4c the FFT amplitude spectra determined from dynamics in the center of the CoFeB film as a function of the external field. This plot clearly reveals a resonance like characteristic, with a maximum at *μ*_0_ *H*_*ex*_ = 600 mT.

## Summary

We have shown how acoustically hard capping layers can be utilized to characterize elastic properties of SLs. From the spectral point-of-view, the approach is limited by the bandwidth of the optically induced thermal stress pulse. As an idealized upper bound, the duration of the laser pulse (60 fs) implies applicability up to 15 THz. If not relying on electronically driven thermal expansion, temperature equilibration between the laser-heated electron gas and the lattice delays the lattice-temperature-driven thermal expansion^29^. Thus it is more realistic to assume a few THz as a practical upper limit. Furthermore, we have demonstrated that the optically generated large local strain amplitudes can be functionalized for magneto-elastic excitation of exchange-dominated spin wave modes.

Note that in many experimental studies in picosecond ultrasonics, spectrally broad elastic pulses are generated by a capping layer, in order to address dynamics in the interior of the underlying sample. Interpreting the spectral content of the time series is then complicated because the measurement signal results from an integration over the depth of the sample, taking into account further Brillouin scattering processes, and the opacity of the sample^30^. Here, the signal of interest is directly generated at the surface. No elaborated simulation or calculation is needed to understand the basic results. In fact, solving Eq. 2 is sufficient to understand at which frequency range the elastic response of the surface layer takes place.

## Methods

For sample growth a custom-developed pulsed-laser depostion system was employed. XRR characterization was performed with a Bruker D8 Discover with Cu–K*α* radiation, TEM imaging with a Philips CM30 at 300 kV. The optical pump-probe setup is based on a regeneratively amplified Ti:Sapphire laser system (RegA 9040 from Coherent), delivering pulses with a repetition rate of 250 kHz, a central wave length 800 nm, and a pulse width 60 fs (full width at half maximum).

## Electronic supplementary material

movie S1

movie S2

movie S3

Supporting information
