# Supplementary material for: Spectral control of elastic dynamics in metallic nano-cavities
Source: Sci Rep. 2017 Sep 6;7:10600. doi: 10.1038/s41598-017-11099-y (PMC5587714; doi:10.1038/s41598-017-11099-y)
Supplement: Supplementary file 4 — Supporting information [file 41598_2017_11099_MOESM4_ESM.pdf]

Supporting information: Spectral control of elastic dynamics in metallic nano-cavities

Henning Ulrichs,[*,†] Dennis Meyer,[†] Florian Döring,[‡,¶] Christian Eberl,[‡,§] and Hans-Ulrich Krebs[‡]

†*I. Physical Institute, Georg-August University of Göttingen, Friedrich-Hund-Platz 1, 37077 Göttingen, Germany*

‡*Institute of Materials Physics, Georg-August University of Göttingen, Friedrich-Hund-Platz 1, 37077 Göttingen, Germany*

¶*Laboratory of Micro and Nanotechnology, Paul Scherrer Institut, CH-5232 Villigen PSI, Switzerland*

§*Physikalisch Technische Bundesanstalt, Bundesallee 100, 38116 Braunschweig, Germany*

E-mail: hulrich@gwdg.de

In this supplement we provide additional details regarding the analytical and numerical modelling. Table 1 summarizes all material parameters used in the analytical and numerical modelling.

Data analysis

Experimental data was analyzed by first subtracting a background phenomenologically modelled by a double exponential function. Then the datasets were artificially enlarged by

	Speed of sound v_L, $\left(\frac{m}{s}\right)$	Density ρ, $\left(\frac{\text{g}}{\text{cm}^3}\right)$	Acoustic Impedance $Z = \rho v_L$, $\left(\frac{\text{MPa}\cdot\text{s}}{\text{m}}\right)$
MgO	8700*	3.58**	31
ZrO_2	6300*	6.12**	38
W	5220[1]	19.30**	101
CoFeB	5527[2]	7.05[2]	39

Table 1. Material parameters. *Values determined from adjusting the analytically calculated band gap position and width to the measured position and width in SL1 and SL2. **Values determined during PLD sample preparation.

zero-padding, in order to obtain a smooth Fourier spectrum. Note that in case of SL1 the original data set covers 500 ps after excitation, in case of SL2 only 100 ps. In both cases, all oscillations have died out at these time instances. The power spectra were computed by first computing autocorrelation coefficients, then multiplying a Hanning-window to the coefficients, and then performing a fast Fourier transform.

Details regarding the analytic model

The boundary conditions at the interfaces mentioned in the article give rise to the following system of equations:

$$\begin{bmatrix} e^{-i\alpha_0 t_0} & -e^{i\alpha_0 t_0} & 0 & 0 & 0 & 0 \\ 1 & 1 & -1 & -1 & 0 & 0 \\ F_{01} & -F_{01} & -1 & 1 & 0 & 0 \\ 0 & 0 & e^{\alpha_1 t_1} & e^{-\alpha_1 t_1} & -1 & -1 \\ 0 & 0 & F_{12} e^{\alpha_1 t_1} & -F_{12} e^{-\alpha_1 t_1} & -1 & 1 \\ 0 & 0 & 1 + F_{12} e^{-iQL} & 1 - F_{12} e^{-iQL} & -e^{-iQL+\alpha_2 t_2} - e^{\alpha_2 t_2} & e^{-\alpha_2 t_2} - e^{-iQL-\alpha_2 t_2} \end{bmatrix} \cdot \begin{pmatrix} A \\ B \\ C \\ D \\ E \\ F \end{pmatrix} = 0$$

where $F_{\mathrm{ij}} = \frac{\rho_{\mathrm{i}} v_{\mathrm{i}}^2 \alpha_i}{\rho_{\mathrm{j}} v_{\mathrm{j}}^2 \alpha_j}$. Here, the index 0 refers to tungsten, 1 to MgO, and 2 to ZrO_2. To proceed, we determine for a given thickness t_{W} the roots of the systems determinant, that is we solve $|M| = 0$ for f and the imaginary part Q'' of the wavenumber $Q = Q' + iQ''$. Note that we find that only for $Q' = \frac{n\pi}{L}$ such roots exist. Figure 1(a) shows an evaluation of $|M|$ for $Q' = \pi/L$ and $t_{\mathrm{W}} = 15\,\mathrm{nm}$. In this case the solution appears at positive $Q'' > 0$. For a thickness of $t_{\mathrm{W}} = 20\,\mathrm{nm}$ the solution appears at $Q'' < 0$ (see Figure1(b)), whereas for $t_{\mathrm{W}} = 45\,\mathrm{nm}$ a solution with again $Q'' > 0$ can be found (see Figure1(c)).

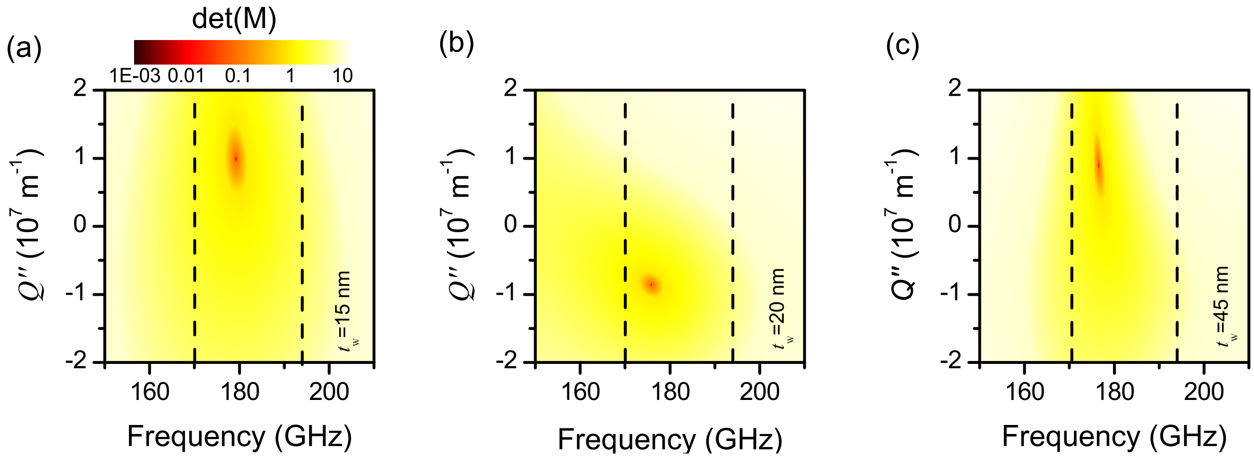

Figure 1: Evaluation of $|M|$ for three different thicknesses of the tungsten layer. For the real part of the wave number, $Q' = \frac{\pi}{L}$ was set. Vertical dashed lines mark the edges of the first band gap of the superlattice in sample SL1.

Details regarding the numerical model

Numerical modelling of light-induced elastic dynamics was carried out with a finite-difference time-domain approach. Our spatially one-dimensional model takes into account the optical absorption of the incident light by means of a transfer-matrix formalism. This absorption leads to a heating of the surface. The temporal evolution of the local temperature is modelled by two coupled diffusion equations. Here, lattice and electron temperatures are distinguished. Heating of the lattice results in thermal expansion, giving rise to a thermal stress pulse. This thermal stress enters the elastic wave equation. The advantage of the numerical model is,

that we can model all important aspects of the experiment, and that it in particular enables us to look inside the sample. As an example, we show in Figure 2 and in the short movie S1 the temporal evolution of the elastic stress in SL1, when excited where the tungsten has a thickness of 15 nm. Note that to reduce the memory amount we have modelled SL1 with only 20 MgO/ZrO_2 double layers. To avoid a large offset in the surface region, we have subtracted the pure thermal stress. In Fig. 2(a) one sees a big initial stress pulse, which travels through the sample. At each interface partial reflections occur. The most prominent reflection event occurs at the interface towards the substrate. Out of the broad spectrum carried by the initial stress pulse, the part, which is inside the band gap around 175 GHz cannot leave the surface. This is exactly the long-living resonance which shows up in the experiment. Time-domain Fourier transforming the data at each point in space allows to represent the data as a color-coded stress map shown Figure 2(b). Here one can directly see the mode profile of the surface resonance at 176 GHz, which perfectly agrees with the analytic calculation. A spatial Fourier transformation of the original data is shown in Figure 2(c). Here, the wave number $Q' = 157 \cdot 10^6\ \mathrm{m}^{-1} = \frac{\pi}{L}$ dominating the dynamics is highlighted. Note that this value lies precisely at the egde of the Brillouin zone, as expected from the theory. Complete spatio-temporal Fourier transformation yields the map shown in Figure 2(d). Note that here a logarithmic color scale is used. This highlights that besides the surface resonance the travelling stress pulse also populates collective resonance modes of the total superlattice. These resonances lie on the dispersion curves for the infinite superlattice.

The FDTD model confirms the findings of the analytical theory. In particular the simulation confirms that if the thickness t_{W} is chosen such that $Q'' > 0$, a strong response in the band gap appears. Moreover, suppression of dynamics if $Q'' < 0$ can be seen in Figure 3, and in the corresponding second movie S2, which show simulated dynamics for the case of $t_{\mathrm{W}} = 20$ nm. In agreement with the experiment no pronounced dynamics associated with the band gap appears. Instead, the weak response around 130 GHz$\approx \frac{v_L}{2t_{\mathrm{W}}}$, lies in the first band. Thus it can leave the surface, and decays fastly.

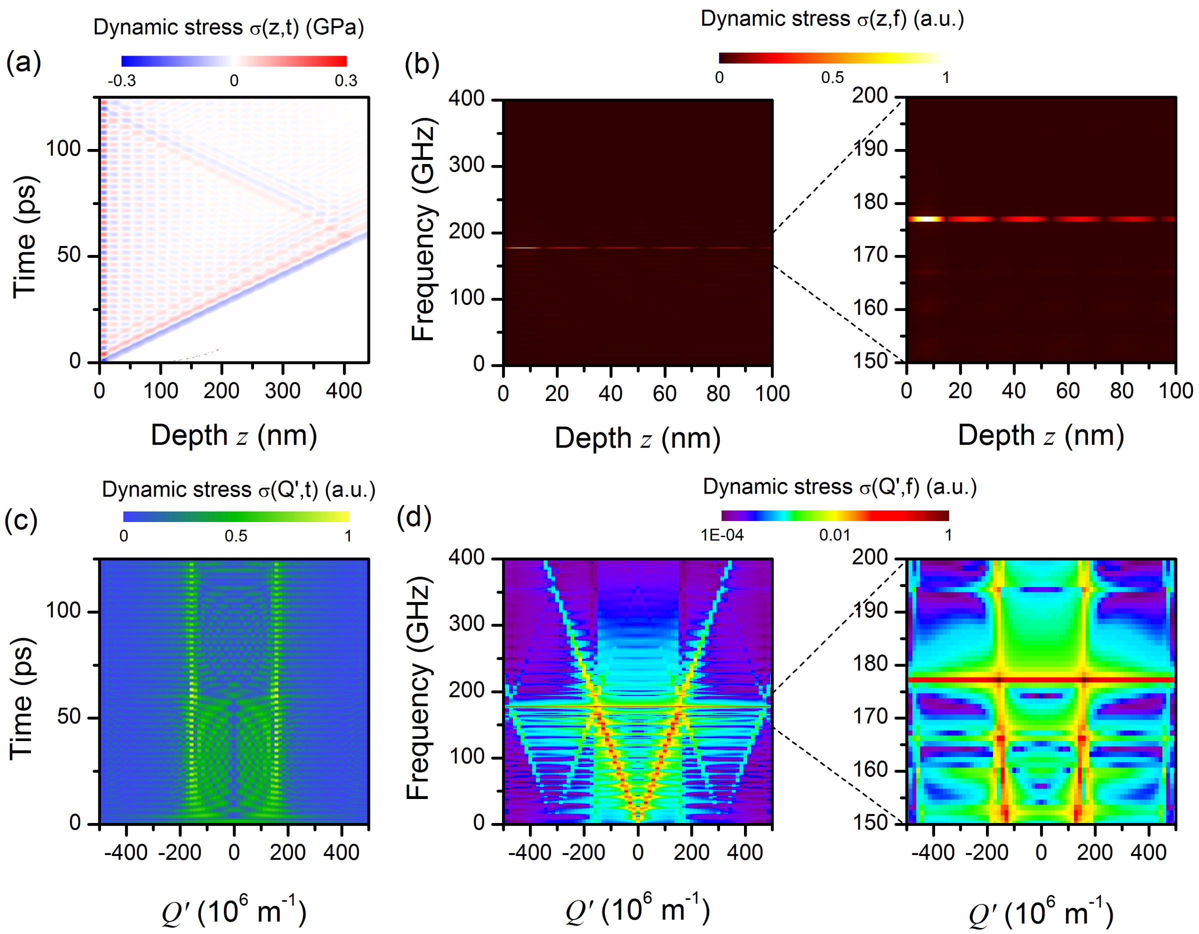

Figure 2: Numerical simulation of light-induced elastic dynamics in sample SL1 at a location where the tungsten layer has a thickness of 15 nm. (a) Elastic strain as function of depth z and time. (b) Time-domain FFT of σ. (c) Spatial FFT of σ. (d) Spatio-temporal FFT of σ.

Recall that the analytic theory predicts that Q'' eventually turns positive again when further increasing t_{W}. We test this analytic prediction numerically by setting the thickness to $t_{\mathrm{W}} = 45\,\mathrm{nm}$. Indeed, the data shown in Figure 4, and the third movie S3, confirm the excitability of a third order resonance mode in the tungsten film.

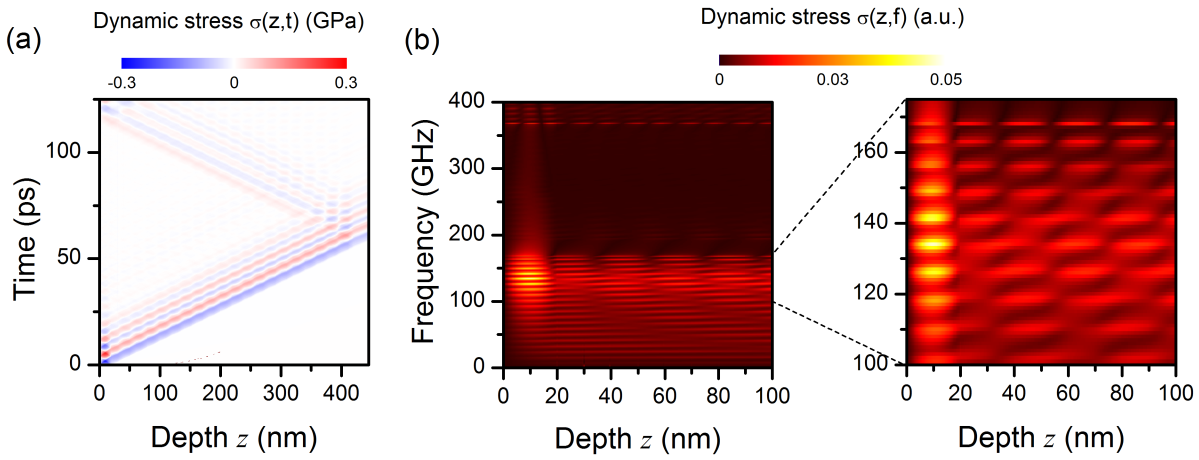

Figure 3: Numerical simulation of light-induced elastic dynamics in sample SL1 at a location where the tungsten layer has a thickness of 20 nm. (a) Elastic strain as function of depth z and time. (b) Time-domain FFT of σ. Note that the color scale covers almost two orders of magnitude less than in Figure 2(b).

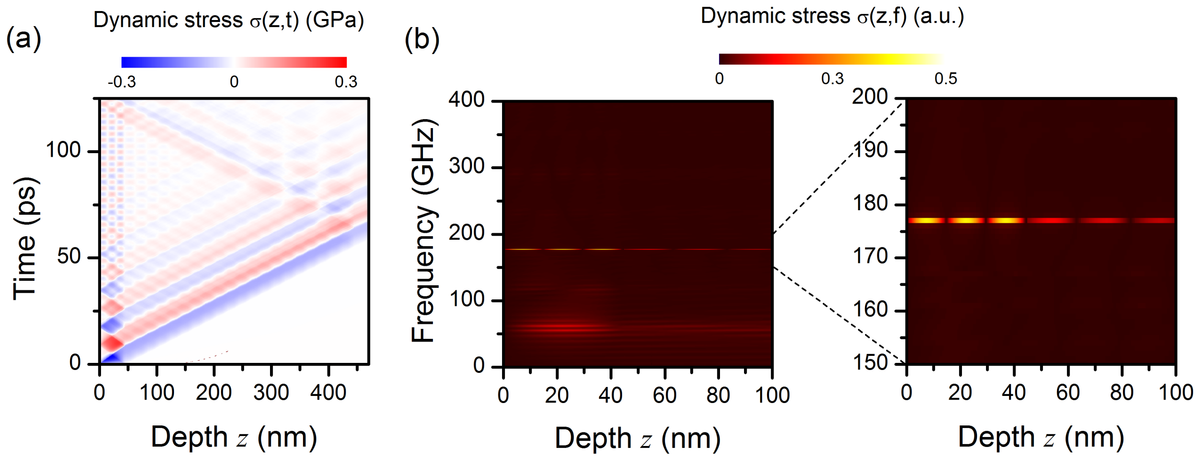

Figure 4: Numerical simulation of light-induced elastic dynamics in sample SL1 at a location where the tungsten layer has a thickness of 45 nm. (a) Elastic strain as function of depth z and time. (b) Time-domain FFT of σ.

References

1. Döring, F.; Major, A.; Eberl, C.; Krebs, H.-U. Minimized thermal conductivity in highly stable thermal barrier W/ZrO2 multilayers. *Applied Physics A* **2016**, *122*, 872.

2. Ulrichs, H.; Meyer, D.; Müller, M.; Wittrock, S.; Mansurova, M.; Walowski, J.; Münzenberg, M. THz elastic dynamics in finite-size CoFeB-MgO phononic superlattices. *Journal of Applied Physics* **2016**, *120*, 142116.
